# How Does an Eating Disorder Affect Your Physical and Mental Health and How It Is Related to Sleeve Gastrectomy?

**DOI:** 10.7759/cureus.50832

**Published:** 2023-12-20

**Authors:** Osman Suliman, Ammar A Alrazehi, Badr A Alsafar, Abdullah A Kaki, Alwaleed A Alsafar, Mazen K E. Alharbi, Mazen K M. Alharbi, Fares A Abed, Asim A Almohammadi, Emad A Alsaedi

**Affiliations:** 1 College of Clinical Sciences, Faculty of Medicine, Al-Rayan Colleges, Madinah, SAU; 2 College of Medicine, Faculty of Medicine, Al-Rayan Colleges, Madinah, SAU

**Keywords:** eating disorder, sleeve gastrectomy, obesity, mental health, physical health

## Abstract

Background

Obesity is the pandemic of this era. At the same time, the commercialisation of thinness has also increased its adversity. Dissatisfaction with body shape is leading to many eating disorders. These disorders further cause several health problems. It has been found that individuals with eating disorders experience insomnia, depression, and anxiety. It also affects the endocrine system and digestive systems of the body. The surgical approach provides a more efficient treatment of obesity with sustainable results. Sleeve gastrectomy is the most popular surgical treatment. Sleeve gastrectomy is one of the prominent bariatric surgeries. Patients going through sleeve gastrectomy not only lose weight but also improve their mental health.

Objective

This study assesses the relationship between obesity, eating disorders, and physical health. It also focuses on the prevalence of sleeve gastrectomy procedures in such individuals and their outcomes.

Methods

This study used a cross-sectional design and a convenient sampling technique. The obese individuals who had eating disorders residing in Medina Al-Munawara and Riyadh city were taken as the samples. Online questionnaires were shared with participants to collect their perspectives on their weight, eating disorders, and their impact on physical health. The study collected both retrospective and present data.

Results

A total sample of 335 participants was taken, of which 181 (54%) were females and 154 (46%) were males, with a mean BMI of 28.85±2.57. The fear of weight gain was found among 265 (79%) of participants, 151 (45%) were feeling guilt while eating, 275 (82%) were weighing themselves daily, 325 (97%) were unable to stop eating even when complete, and 117 (35%) were unable to control themselves when they have food. As per the methods of avoiding weight gain, 166 (49.6%) were skipping a meal, 157 (47%) were following a diet, 17 (5%) were inducing vomiting, and 16 (4.8%) reused laxatives and diuretics. A total of 158 (47.2%) were involved in sports, but it was reduced to 61 (18.25%) upon sickness. Only 24 (7%) participants had undergone sleeve gastrostomy, and 181 (54%) believed that their cravings had increased, 238 (71%) complained of dizziness, and 151 (45%) believed that fast food caused gut unrest.

Conclusion

Obese individuals who are going through eating disorders are psychologically and physically compromised. They consider skipping meals to be the most efficient means of weight loss, and only a small percentage prefer to go for sleeve gastrectomy.

## Introduction

Eating disorders (ED) are categorised as psychiatric disorders that lead to abnormal eating patterns. There could be multiple causes that lead to ED, but one of the most prevalent is body shaming, wherein people make negative comments about an individual's body shape or size. It is predominantly found in adolescents and youngsters as they are attracted more toward society’s standard of being beautiful. In a cross-sectional study performed on college students in America, results showed that ED was more common in females than males. Another study finding was that EDs were more prevalent in overweight and obese individuals [1].

Anorexia nervosa is an ED categorised as the most life-threatening psychiatric disorder. The mortality rate is pretty high in the case of anorexia nervosa. In this ED, the patient stops eating in fear of getting obese, and it leads to severe malnutrition and muscle wasting [2].

Bulimia nervosa is just the opposite of anorexia nervosa as individuals with this psychiatric disorder go for overeating [3]. Another category is ED, which is not specified, such as binge eating. Binge eating is when an individual eats a large portion of a meal and then feels guilty. Feeling guilty makes him throw the food out by self-induced vomiting or by taking diuretics and laxatives. A person is categorised as binge eating if the frequency is twice a week for six months.

As the prevalence of obesity is increasing day by day, people are trying to get rid of extra pounds without lifestyle modifications. Hence, the prevalence of surgical treatments for obesity and EDs is increasing day by day. Out of all bariatric surgeries, sleeve gastrectomy is the most famous one. In this procedure, a part of the stomach is closed so that less food can enter the gut. These procedures somehow show rapid weight loss results, and the incidence of obesity-related co-morbidities is lowered in the long run [4].

Individuals are found to lose significant weight after surgery, and a significant improvement is seen in the quality of life and depression status of obese patients, though the linkage still needs to be developed [5]. Another study compared gastric banding with sleeve gastrectomy. It concluded that sleeve gastrectomy improves food tolerance and eating behaviors. The surgical procedure showed improvement in all fields of the quality-of-life questionnaire, other than mental health [6].

Obesity is a health problem, and its prevalence in Saudi Arabia is being studied. According to a study conducted in 2020, 24.7% of Saudi individuals are obese (BMI>30) [7], whereas it was more in 2018 as 25.6% of Saudis were obese then, but in 2013, 28.7% were obese. Hence, there is improvement in the prevalence of obesity, but, still, a significant number must be catered to. The socio-cultural advancement in the Arab region has also increased body shaming. The status of thinness as an ideal has also been appraised in the Arab world, and many females are affected by it, leading to ED [8].

EDs affect the gut health and cause many other health problems. Such psychiatric condition affects the behavioral and mental state of an individual. The neurological co-morbidities with ED are depression, sleep disturbances, bipolar disorder, and suicidal thoughts. When ED results in such mental health disturbances, it leads to malnutrition and physical health problems. In ED, people do not take enough or do not give food enough time to be absorbed in the gut, resulting in many malnutrition issues. It dehydrates a person, further affecting general GI health and causing abdominal cramps, osteoporosis, cardiovascular diseases, endocrine hormonal imbalance, and skin problems [9].

The severity of EDs is lowered after bariatric surgery. Many individuals undergoing sleeve surgeries later show better results, but the linkage between sleeve surgeries and ED is still unknown [10]. Thus, the current study investigates the association of ED with Saudi individuals' physical and mental health and its link with sleeve gastrectomy.

The objectives of this study are to assess the relationship between obesity, EDs, and physical health of obese individuals. Specifically, the study aims to examine the impact of EDs on physical health and explore the prevalence of sleeve gastrectomy in such patients.

## Materials and methods

Study design

A cross-sectional study design was followed in the current study. The sampling was done through convenient selection. Data were collected from the subjects who were obese and were suffering from EDs.

Study setting

The study took place in Al Medina Al-Munawara and Riyadh City of Saudi Arabia, where there are many specialised centres for sleeve gastrectomy.

Period

Data were collected and analysed for the present study from February 2023 until November 2023.

Study population/subjects

Saudi Arabs, both males and females, living in Medina Al-Munawara and Riyadh, who are obese and have any ED as per the inclusion and exclusion criteria, were the population under study, as mentioned in Table 1.

**Table 1:** Gastrectomy as per the inclusion and exclusion criteria

Inclusion Criteria	Exclusion Criteria
Candidates with morbid obesity and suffering from ED	Non-obese candidates
Especially morbid obesity related to eating disorders in AL Medina Al-Munawara region and Riyadh City	The candidates who underwent sleeve gastrectomy for causes other than eating disorders

Sample size

The total population of Saudi Arabia is around 21,690,648. Based on the prevalence of obesity in Saudi Arabia, it is 24.7%, so it will make a population of three million two lac Saudis. A report suggests that 30,000 individuals go for sleeve gastrectomy annually in Saudi Arabia [11]. This study gathered data from 270 individuals (as calculated from Qualtrics with a confidence interval of 90%) living in Saudi Arabia who visited bariatric surgery centers for consideration of sleeve gastrectomy because of their EDs.

Sampling technique

A convenient sampling technique was used in the current research.

Data collection methods, instruments used, and measurements

An online questionnaire was shared with the study participants. The questionnaire was developed based on the questions made by the Eating Disorder Foundation [12]. The patients were asked to fill in data keeping in view their eating behaviors and their strategy to tackle the issue. Further questions were asked to relate to their physical health. To keep the answers close, an ended 5-point Likert scale was used to collect responses.

Data management and analysis plan

Data were recorded in Excel and the help of Statistical Product and Service Solutions (SPSS, BM SPSS Statistics for Windows, Armonk, NT) analysis. Demographic details were analysed by descriptive analysis on SPSS, and then the association between psychological stress and health was measured by Pearson's chi-square test. A value of 0.05 was considered the value of statistical significance for all statistical tests.

Ethical considerations

Ethical approval for conducting the study was taken from the Al-Rayan Research Ethics Committee (registered with the National Bioethics Committee in KACST, Saudi Arabia). The study ID was HA-03-M-122-021, dated 25/2/2023. Participants were verbally informed of the details of the research purpose ahead of time, and their consent was taken. Questions were asked, and approval was taken in Arabic language. They were independent in deciding if they wanted to fill out the form. The personal data of participants were kept confidential.

## Results

Descriptive analysis of data

Age Distribution of Participants

**Figure 1:**
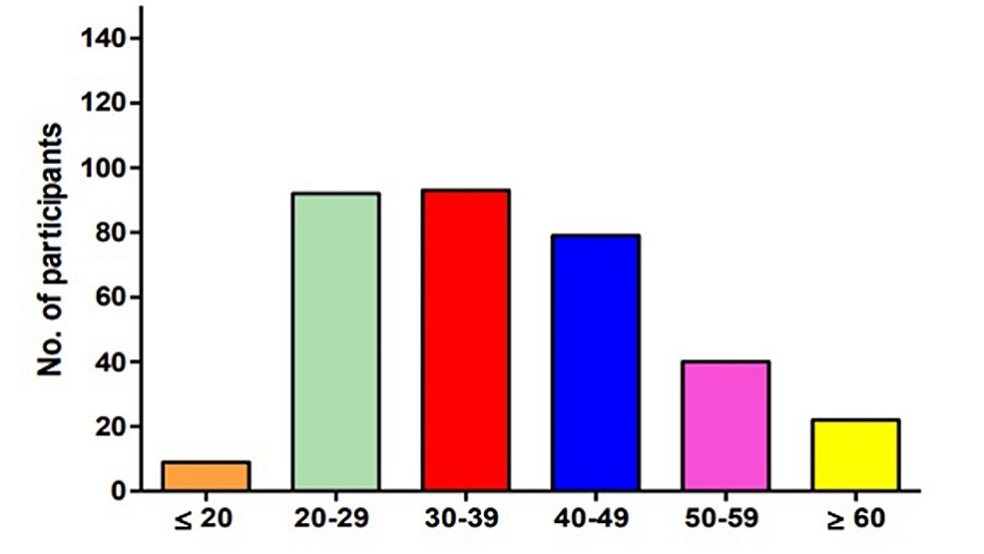
Age distribution of data The data have been represented as the percentage (%) of occurrence of the variables.

A total sample of 335 participants was taken under study. The ages of participants were divided into six groups. As per the calculated data, 2.7% (9) of the participants were from age less than 20 years, 27.5% (92) of participants were aged 20-29 years, 27.7% (93) of participants were aged 30-39, 23.6% (79) of participants were aged 40-49 years, 11.9% (40) of participants were aged 50-59 years years, and 6.6% (22) were of age more than 60 years. Frequencies and percentages of age distribution are summarised in Figure 1.

Gender Distribution of Participants

In the current study, 46% (155) of participants were male, whereas 54% (180) were females. A summary of gender distribution can be seen in Table 2.

**Table 2:** Gender distribution of participants The data have been represented as the frequency and percentage (%) of occurrence of the variables.

Gender	Frequency	Percentage
Male	155	46%
Female	180	54%
Total	335	100%

Anthropometric Measurements of Participants

The participants were also asked about their weight and height in demographic data. The mean values and BMI were calculated based on participants’ reported weight and size. The mean body weight of participants was 77.25±20.66 kg, the mean height was 163.66±12.87 cm, and the calculated mean BMI of participants was 28.85±2.57. Data can be seen in Table 3.

**Table 3:** Anthropometric measurements of participants The data have been represented as per the mean value of correlation among the variables.

Anthropometric Measurements	Mean+Std
Weight	77.25±20.66 kg
Height	163.66±12.87 cm
BMI	28.85±2.57

Behaviors in EDs

**Figure 2:**
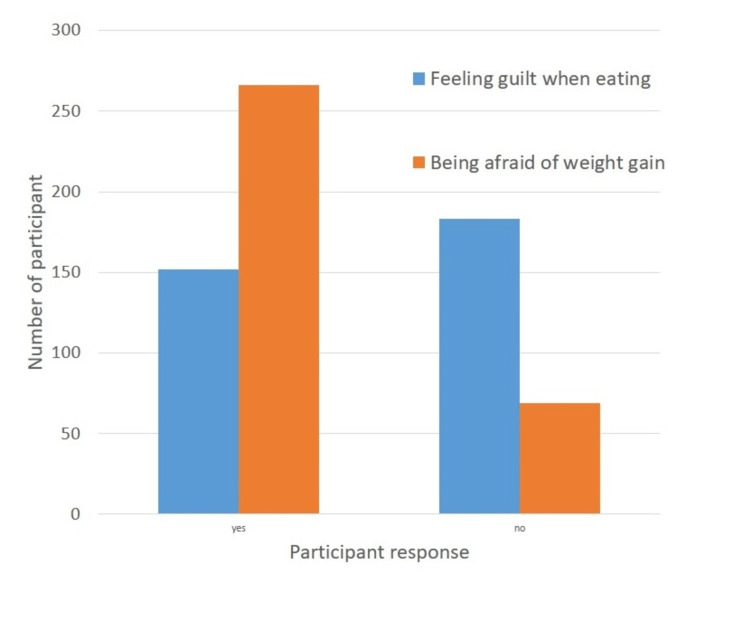
Symptoms of eating disorders

**Figure 3:**
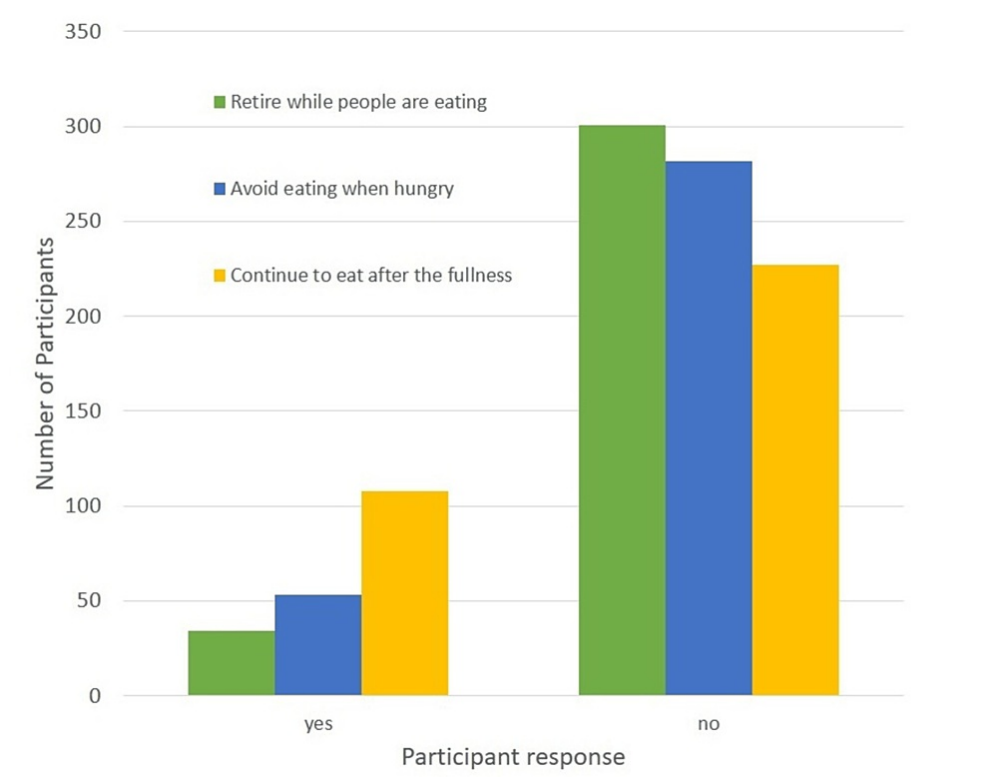
Symptoms of eating disorders

In the second section of the questionnaire, participants were asked to evaluate if they had any ED symptoms. Out of the 335 participants, 45.1% (152) responded that they feel guilt when eating, 79% (266) were afraid of weight gain, 10.1% (34) said they retire when people are even eating, 15.8% (53) avoid eating even when hungry, only 3% (10) reported using medicines as replacement of meals, 35.8% (120) said that they are feeling out of control when they see food, 82.1% (275) of them weighed every day, and 32.2% (108) of participants reported that they keep eating even if they are full. Data are summarised in Table 4 and Figures 2-3.

**Table 4:** Symptoms of eating disorders The data have been represented as the frequency and percentage (%) of occurrence of the variables.

Symptoms of Eating Disorders	Yes	No	Total
Feeling guilt when eating	152 (45.1%)	183 (54.6%)	335 (100%)
Being afraid of weight gain	266 (79%)	69 (21%)	335 (100%)
Retire while people are eating	34 (10.1%)	301 (89.9%)	335 (100%)
Avoid eating when hungry	53 (15.8%)	282 (84.2%)	335 (100%)
Take medication instead of a meal	10 (3%)	325 (97%)	335 (100%)
Feel out of control while eating	120 (35.8%)	215 (64.2%)	335 (100%)
I weigh myself daily	275 (82.1%)	60 (17.9%)	335 (100%)
Continue to eat after the fullness	108 (32.2%)	227 (67.8%)	335 (100%)

Methods to avoid weight gain

Further in detail, participants were asked what methods they use to avoid weight gain. Four options were given to the participants, according to which 47.5% (159) used diet regimes, 49.6% (166) skipped a meal, 4.8% (16) used laxatives, and 5% (17) induced vomiting after eating. Data are summarised in Table 5.

**Table 5:** Methods of avoiding weight gain The data have been represented as the frequency and percentage (%) of occurrence of the variables.

Methods of preventing weight gain	Yes	No	Total
Diet regime	159 (47.5%)	176 (52.5%)	335 (100%)
Skip a meal	166 (49.6%)	169 (50.4%)	335 (100%)
Use diuretics and laxatives	16 (4.8%)	319 (95.2%)	335 (100%)
Induce vomiting	17 (5%)	318 (95%)	335 (100%)

Distribution of participants based on sports activities

Current study data were taken online, so the participants were asked if they were involved in sports activities. Of 335 participants, 47.2% (158) responded that they involve themselves in sports. On asking if they continue the sports even when tired or sick, only 18.2% (61) answered yes. Data are summarised in Table 6.

**Table 6:** Sport activities The data have been represented as the frequency and percentage (%) of occurrence of the variables.

Physical activity	Yes	No	Total
Practice sports	158 (47.2%)	177 (52.8%)	335 (100%)
Practice sports when feeling tired or sick	61 (18.2%)	274 (81.8%)	335 (100%)

Prevalence of sleeve operation

**Figure 4:**
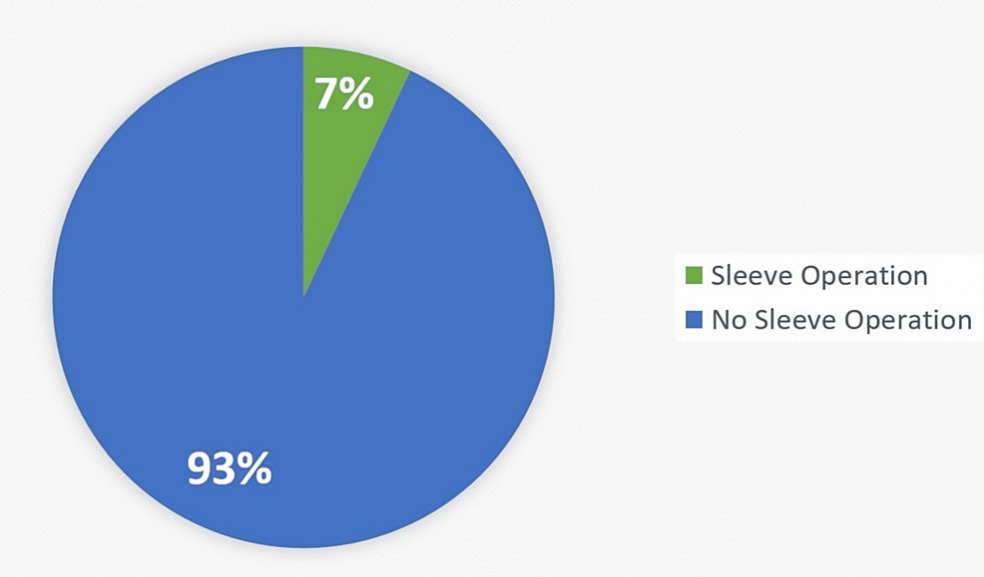
Prevalence of sleeve operation The data have been represented as the percentage (%) of occurrence of the variables.

To find the prevalence of participants that go through sleeve operation to avoid weight gain, participants were asked about their history of the procedure; only 24(7%) reported having an account of sleeve operation. Data are summarised in Figure 4.

Eating behaviors of participants with sleeve gastrectomy

The participants who had the sleeve procedure history were further asked about their eating behaviors. Their answers were collected on a number scale, ranging from 0 to 5, in which 0 meant the least and 5 told the most. This section asked three questions related to hunger, appetite, and pleasure levels with meals. The mean value for hunger level was 2.75±1.36; for appetite level, 2.91±1.41; and, for pleasure level, 2.63±1.5. Data are summarised in Table 7.

**Table 7:** Eating behaviors after sleeve gastrectomy The change in eating behavior was scaled from 0 to 5 in which the frequencies and percentage of responses are shown in the table, along with the mean values.

Eating Behaviors	0	1	2	3	4	5	Mean±Std
Hunger level	1 (4.2%)	3 (12.5%)	6 (25%)	9 (37.5%)	1 (4.2%)	4 (16.7%)	2.75±1.36
Appetite level	0	4 (16.7%)	7 (29.2%)	5 (20.8%)	3 (12.5%)	5 (20.8%)	2.91±1.41
Pleasure level	1 (4.2%)	6 (25%)	5 (20.8%)	4 (16.7%)	5 (20.8%)	3 (12.5%)	2.63±1.5

Supplementation and food cravings after sleeve gastrectomy

Vitamin B12 is considered an essential supplementation required after the procedure. Upon asking about the consumption of vitamin B12, only 50% (12) of participants responded favorably. Participants were also asked if they craved food more, and 54% (13) answered yes, and most were craving fast food. Data are summarised in Table 8.

**Table 8:** Supplementation and food craving The data have been represented as the frequency and percentage (%) of occurrence of the variables.

Behaviors and practices	Yes	No	Total
Vitamin B12 supplement	12 (50%)	12 (50%)	24 (100%)
Craving fast food	13 (54%)	11 (46%)	24 (100%)

Sleeve gastrectomy and dizziness 

**Figure 5:**
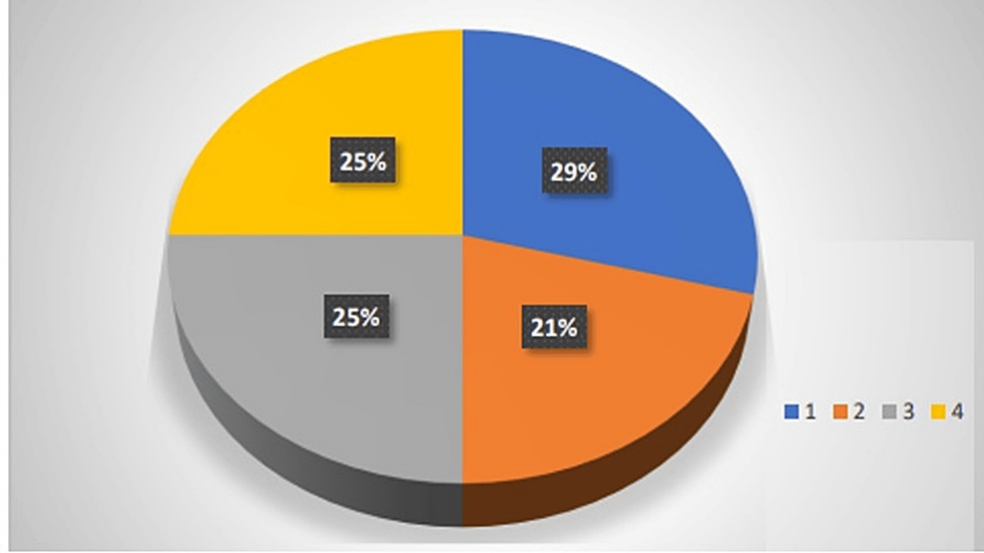
Occurrence of dizziness in the participants Here, code 1 represents the participants who do not feel any dizziness, code 2 represents the participants who feel dizzy in the morning, code 3 represents the participants who feel dizzy before a meal, and code 4 represents the participants who feel dizzy after a meal.

To evaluate participants’ mental health, they were asked if they felt dizzy at times after the sleeve gastrectomy, and 70.8% (17) responded yes. Then, participants were asked the times of day they mainly felt dizzy. Seventeen participants responded the following: 21% (five) in the morning, 25% (six) before meals, and 25% (six) after meals, and the remaining 29% (seven) did not feel any dizziness. Data are summarised in Figure 5.

Foods that cause unrest

**Figure 6:**
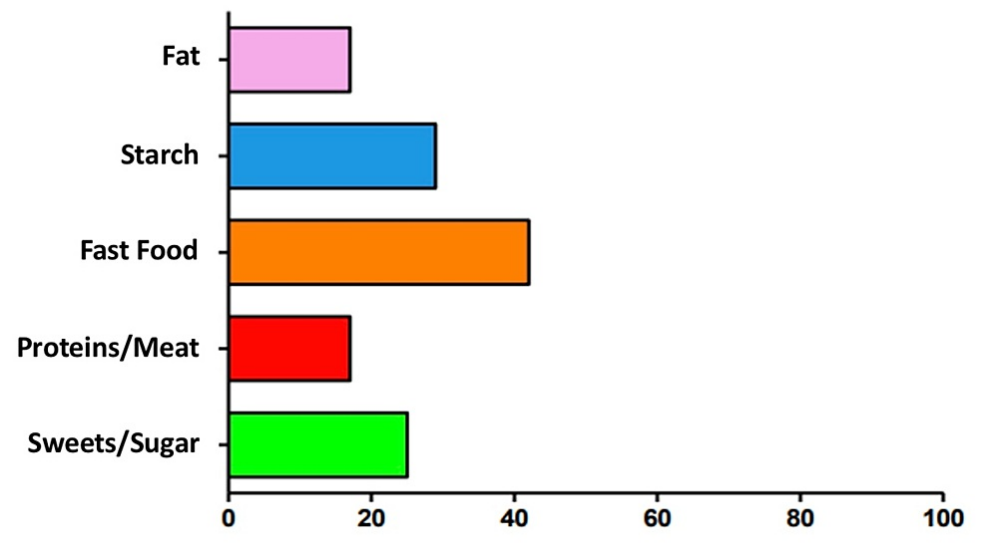
Foods causing gut problems The data have been represented as the percentage (%) of occurrence of the variables.

Lastly, the participants were asked about foods that cause abdominal stirs, such as diarrhea, bloating, and nausea, after sleeve gastrectomy. There were five options given to participants. According to the participants, 45% (11) believed fast food causes gut problems, 30% (seven) thought it is starch, 35% (eight) believed it is sweets and sugars, 18% (four) thought it is fat, and the last 18% (four) thought it was protein according to participant perception. Data are summarised in Figure 6.

Association between physical health factors in sleeve operation participants

A bivariate correlation test was applied to see if there was any significant link between physical and mental concerns in participants. It was seen that hunger, appetite, and pleasure while eating are substantial (p<0.01), whereas other factors, such as vitamin B12 intake, cravings, and dizziness, are not significantly associated. Data are summarised in Table 9.

**Table 9:** Association between physical health factors in sleeve operation participants **Correlation is significant at the 0.01 level (2-tailed).

Correlations		Vitamin B12	Hunger	Appetite	Craving
Pleasure	Pearson Correlation	0.256			
	Sig. (2-tailed)	0.228			
	N	24			
Hunger	Pearson Correlation	0.188			
	Sig. (2-tailed)	0.379			
	N	24			
Appetite	Pearson Correlation	0.121	0.940^**^		
	Sig. (2-tailed)	0.575	0.000		
	N	24	24		
Craving	Pearson Correlation	0.084	0.016	0.116	
	Sig. (2-tailed)	0.698	0.942	0.589	
	N	24	24	24	
Dizziness	Pearson Correlation	0.036	0.007	0.028	0.003
	Sig. (2-tailed)	0.867	0.975	0.896	0.989
	N	24	24	24	24

## Discussion

Obesity is a pandemic that is affecting the whole world. It is a multifactorial health concern that leads to many non-communicable diseases. Other than health concerns, it leads to body shaming, so people try to keep their weight as per the ideal body weight standards. Being concerned about weight and looks sometimes leads to EDs where the patient feels guilt and remorse if he eats and tries to induce vomiting. EDs are psychological health problems. There are eight recognised "feeding and eating disorders," according to the most recent Diagnostic and Statistical Manual (DSM-5): pica, rumination disorder, avoidant/restrictive food intake disorder, anorexia nervosa, bulimia nervosa, and binge-eating disorder [13]. The current study was planned to find the occurrence of EDs among Saudi inhabitants, mainly from Madinah Munawerah and Riyadh and to find the link between EDs with physical and mental health. 

There were 335 participants under study: 45% (151) were preoccupied with fear and felt guilty about eating, 79% (265) were afraid of weight gain, and 10% (33) reported leaving when others were eating. These stats show that a majority of participants had the symptoms of EDs. These symptoms are not enough to diagnose but refer to a person’s psychiatric state, which can lead to a severe ED [14]. Another prominent finding was that 82% (275) of participants weighed themselves daily. Based on EDE Q scaling, the guilt of eating, social eating, and losing control over eating all come under eating concerns; in this study, approximately 50% (167) of individuals are having problems with their eating concerns. According to the ED examination, losing control of eating refers to a bulimic episode; 35% (117) of participants in this study reported that they lose control while eating, and 32% (107) stated that they keep on eating even when they are packed, so it refers to bulimic ED [15]. Avoiding food even when hungry is an eating concern that refers to an anorexic episode, and 15.8% (53) of participants responded yes to such a state. According to a study previously conducted in Saudi, bulimia, binge eating, and anorexia were 3.2% within 12 months, and the lifetime prevalence was 6.1% [16]. Further, the participants were asked about the methods that they follow to avoid weight gain; this is a part of the restraint concern. Specifically, 47.5% of participants followed a diet, and 49% preferred to skip meals. Other than that, only 5% were inducing vomiting, which refers to binge eating.

As per the objective of a research study, the effect of EDs on physical and mental health was investigated: 47.2% responded yes when asked if they indulge in sports, and 18.2% responded positively when asked if they follow their routine even when sick or tired. This shows that EDs can negatively impact individuals' physical health. A similar study on adolescents found that EDs’ effect on physical and mental health is non-negotiable. It states that following age, sex, socioeconomic status, co-occurring psychiatric disorders, adolescent health issues, body mass index, and worries about health during adulthood, adolescents with EDs were at significantly higher risk for anxiety disorders, cardiovascular symptoms, chronic fatigue, chronic pain, depressive disorders, and limitations in activities due to poor health, infectious diseases, insomnia, neurological symptoms, and suicide attempts during early adulthood. Regardless of whether an eating problem had been present, issues with eating or weight during adolescence predicted poor health outcomes in adulthood [17]. Studies have suggested improving physical activity in ED patients as they have neurological impacts, which can enhance patient psychiatric and ED conditions [18]. 

Bariatric surgery, especially sleeve gastrectomy, is becoming popular daily to control eating behaviours and weight. In the current study, 7% (24) of participants had undergone sleeve operations. Their eating behaviours and mental concerns were evaluated to find the impact of surgery on EDs. As per their hunger, appetite, and pleasure levels while eating, the values were entirely satisfactory on a scale of 0-6. The score was between 2 and 4, which means their eating pattern is moderate. Their hunger levels and appetite levels were not compromised. Supporting the findings of this study, research was conducted in which the eating behaviours of patients were studied at intervals of 6, 12, and 24 months. It was seen that the food choices had improved over time, and patients had shifted towards healthy eating [19]. In the current study, 54% of participants said that their cravings have increased after the procedure and, most prominently, for fast foods. This refers to the fact that the impact can be short term. These food cravings should be controlled if not on time; these can refer to mental and behavioural health problems [20]. The mental health after sleeve gastrectomy was evaluated based on dizziness suffered by 70% of participants. Participants suffered dizziness before meals (25%), after meals (25%), and in the morning (21%). A case study of a 42-year-old lady also states that, after a sleeve gastrectomy, she complained of vertigo and unilateral hearing loss. The literature supports that dizziness can be a common complaint of patients after the procedure. The Eustachian tube malfunction brought on by muscular relaxation may explain these issues. The decrease of adipose tissue around the ear muscles may lead to muscular relaxation in and of itself [21]. A few other concerns related to complications of sleeve gastrectomy have also been studied, as it has been reported that weight loss after the surgery is linked with nutrient deficiencies too [22]. Another risk factor that has been studied is the chance of postoperative staple line leakage (PSLL) after sleeve gastrectomy; though the prevalence is not high, the management should be communicated before the procedure [23].

Lastly, participants were asked which food groups are the most uncomfortable for the gut, causing nausea, vomiting, diarrhoea, or constipation. Most prominently, participants highlighted fast food and sugary stuff. Studies suggest that bariatric surgery is linked to a considerable decrease in food cravings and consumption, except for high-fat foods. The role of a particular food group is yet to be studied. 

This study focuses on the impact of an ED on physical and mental health, specifically in relation to sleeve gastrectomy, within the cities of Madinah and Riyadh. Data were collected through an online questionnaire, thereby acknowledging the need to consider the local context regarding respondent understanding and potential bias. The sample size in this study is too small to generalise the result nationwide. This study should be conducted on a more significant scale. Future studies should aim for larger samples and explore long-term effects using longitudinal study designs, taking into account the unique sociocultural aspects of the local context.

## Conclusions

This cross-sectional study concluded that most obese individuals with EDs try to control their food intake to reduce weight. There was a considerably higher percentage of individuals who were not eating even when hungry, which can lead to anorexia nervosa, and a small percentage of them were inducing vomiting, which can lead to binge eating. Furthermore, only 7% of the participants went for sleeve gastrectomy, which shows that there is still a small number of individuals in Saudi Arabia who prefer surgical treatment.
